# Preprocedural systemic immune-inflammation index predicts atrial fibrillation recurrence after catheter ablation: A systematic review and meta-analysis

**DOI:** 10.17305/bb.2026.13614

**Published:** 2026-02-04

**Authors:** Bingshan Zhang, Shourong Lu, Zhehao Yin, Kaicheng Wang

**Affiliations:** 1 Department of Geriatrics, The Affiliated Huishan Hospital of Xinglin College, Nantong University, Wuxi, China; 2 Department of Geriatrics, The Affiliated Wuxi People’s Hospital of Nanjing Medical University, Wuxi, China

**Keywords:** Systemic immune-inflammation index, atrial fibrillation, catheter ablation, recurrence, meta-analysis

## Abstract

Inflammation plays a significant role in the pathophysiology of atrial fibrillation (AF) and may affect the likelihood of AF recurrence following catheter ablation. The systemic immune-inflammation index (SII), calculated from circulating neutrophils, lymphocytes, and platelets, has emerged as a promising inflammatory biomarker. This meta-analysis aimed to assess the relationship between preprocedural SII and the recurrence of AF post-ablation. We conducted comprehensive searches across PubMed, Embase, Web of Science, Wanfang, and China National Knowledge Infrastructure (CNKI) for longitudinal observational studies reporting the correlation between preprocedural SII and AF recurrence after either radiofrequency or cryoballoon ablation. Risk ratios (RRs) were aggregated using random-effects models to account for heterogeneity. A total of ten cohort studies involving 4,045 patients were included in the analysis. Our findings indicate that a high preprocedural SII is significantly associated with an increased risk of AF recurrence (RR = 2.32, 95% CI 1.68–3.21; I^2^ ═ 86%). This association remained robust across sensitivity analyses (RR range 2.07–2.53) and showed consistency across predefined subgroups based on sample size (<400 vs. ≥400), age (<61 vs. ≥61 years), sex distribution (<60% vs. ≥60% men), SII cutoff (<510 vs. ≥510), ablation modality (RFCA vs. CBA), follow-up duration (<20 vs. ≥20 months), and study quality (all *P* for subgroup differences >0.05), although these subgroup analyses were exploratory in nature. Meta-regression did not reveal significant study-level modifiers. Additionally, a further meta-analysis treating SII as a continuous variable demonstrated that each 100-unit increase in SII correlates with a higher recurrence risk (RR = 1.09, 95% CI 1.04–1.13; I^2^ ═ 43%). In conclusion, elevated preprocedural SII is associated with an increased risk of AF recurrence after catheter ablation, indicating that SII may serve as a potential adjunctive marker of inflammatory status, pending further prospective validation.

## Introduction

Atrial fibrillation (AF) is the most prevalent sustained cardiac arrhythmia globally, affecting over 37 million individuals and significantly contributing to morbidity, mortality, and healthcare costs [1, 2]. Its prevalence continues to escalate due to aging populations and increasing cardiovascular risk factors [3]. Catheter ablation—utilizing either radiofrequency catheter ablation (RFCA) or cryoballoon ablation (CBA)—has become a recognized rhythm-control therapy for symptomatic AF, demonstrating superior efficacy compared to antiarrhythmic medications in maintaining sinus rhythm and enhancing quality of life [4, 5]. Nevertheless, despite advancements in technology, 20%–40% of patients experience AF recurrence following an apparently successful ablation procedure [6]. This recurrence is clinically significant as it is linked to persistent symptoms, recurrent hospitalizations, additional ablation procedures, and ongoing thromboembolic risks [7]. Therefore, identifying reliable and accessible predictors of post-ablation recurrence is crucial for individualized risk stratification, patient counseling, and the optimization of peri-procedural management strategies [8].

Systemic inflammation plays a pivotal role in the initiation, perpetuation, and structural remodeling of AF [9, 10]. The systemic immune-inflammation index (SII), calculated as platelet count multiplied by neutrophil count divided by lymphocyte count, integrates three primary circulating inflammatory and immune components and has emerged as a robust biomarker in various cardiovascular and metabolic conditions [11, 12]. Mechanistically, elevated SII may facilitate AF recurrence by promoting atrial inflammation, oxidative stress, endothelial dysfunction, and electrical or structural remodeling [13, 14]. In recent years, multiple studies have evaluated preprocedural SII as a predictor of AF recurrence post-catheter ablation; however, findings have been heterogeneous, constrained by modest sample sizes, varying cutoff values, and inconsistent adjustments for confounders [15–24]. Some studies indicate that higher SII correlates with a significantly increased risk of recurrence [15, 16, 19–21, 23, 24], while others report attenuated or nonsignificant associations [17, 18, 22]. To date, no comprehensive synthesis has quantitatively summarized the evidence or explored sources of variability between studies. To address this gap, we conducted a systematic review and meta-analysis of longitudinal observational studies to evaluate the association between preprocedural SII and AF recurrence following catheter ablation and to examine potential modifiers through subgroup and meta-regression analyses.

## Material and methods

The conduct and reporting of this meta-analysis adhered to the PRISMA 2020 recommendations [25] and relevant guidance from the Cochrane Handbook [26], encompassing protocol development, data collection, statistical synthesis, and presentation of findings. The protocol was preregistered with PROSPERO (CRD420251240376).

### Database search

Eligible studies were identified through a comprehensive literature search of PubMed, Embase, Web of Science, Wanfang, and the China National Knowledge Infrastructure (CNKI), employing a broad set of predefined search terms, including: (1) “systemic inflammation index” OR “systemic immune-inflammation index” OR “SII” OR “systemic immune-inflammatory index” OR “systemic-immune-inflammation index”; (2) “atrial fibrillation” OR “AF”; and (3) “ablation” OR “catheter” OR “radiofrequency” OR “RFCA” OR “cryoballoon” OR “pulmonary vein isolation” OR “PVI” OR “recurrence.” The search was limited to human studies and full-text articles published in peer-reviewed journals. Grey literature sources and trial registries were not included, as this review focused solely on peer-reviewed full-text publications. No language restrictions were imposed. To supplement the electronic search, reference lists of relevant original studies and reviews were manually examined to identify additional eligible publications. All databases were searched from their inception through November 9, 2025. Detailed search strategies for each database are provided in Supplemental File 1.

### Study inclusion and exclusion

Study eligibility was defined according to the PICOS framework:

- P (Population): Adult patients (≥ 18 years) with AF undergoing catheter ablation (radiofrequency or cryoballoon), including first-time or repeat procedures and all AF subtypes (paroxysmal, persistent, long-standing persistent).
- I (Exposure): Preprocedural SII, calculated as platelet count multiplied by neutrophil count divided by lymphocyte count. SII was measured before the procedure, either at admission for ablation or during pre-ablation evaluation. Exposure was reported as categorical (high vs. low SII) or continuous (per unit or per standardized increment), with cutoff definitions for high SII consistent with those used in original studies.
- C (Comparator): Patients with lower SII values (reference group) or the non-increment category in continuous analyses.
- O (Outcome): Recurrence of AF after catheter ablation, defined as AF, atrial tachycardia (AT), or atrial flutter (AFL) lasting more than 30 s occurring after the 3-month blanking period. Only studies with follow-up durations exceeding 3 months were considered.
- S (Study Design): Longitudinal observational studies, including cohort studies, nested case-control studies, and post-hoc analyses of randomized or prospective cohorts.

Studies were excluded if they did not evaluate preprocedural SII, did not report post-ablation AF recurrence, lacked an appropriate comparator, or had follow-up durations ≤ 3 months. Studies assessing SII only after ablation, using inflammatory indices without extractable SII values, or not reporting effect estimates or sufficient data for calculation were excluded. Cross-sectional studies, case reports, reviews, conference abstracts, editorials, and non-human studies were also excluded. Studies examining AF occurrence rather than recurrence after ablation were not eligible. If multiple articles derived from the same underlying cohort, only the version with the most extensive dataset or the largest cohort was included.

### Study quality evaluation

Two reviewers (BZ and SL) independently conducted the literature search, study screening, quality appraisal, and data extraction, with any disagreements resolved through consultation with the corresponding author, who was independent of the initial screening and extraction processes and served as an adjudicator. Retrieved records were imported into EndNote X4 (Clarivate Analytics) for reference management and duplicate removal. Title/abstract screening and full-text assessment were conducted independently and in a blinded manner, with reviewers unaware of each other’s decisions. Study quality was assessed using the Newcastle–Ottawa Scale (NOS) [27], which evaluates selection, adjustment for confounding, and outcome assessment, yielding scores from 1 to 9, with higher scores indicating greater methodological rigor. Studies scoring ≥ 7 were classified as high quality.

### Data collection

Extracted data included study-level information (author, publication year, design, and country), participant characteristics (number of patients with AF, mean age, sex distribution, proportion of patients with paroxysmal AF [PaAF], and mean left atrial dimension [LAD] at baseline, and ablation methods), exposure characteristics (timing of SII evaluation, methods of analysis [categorical or continuous variable], and cutoff values for defining a high SII), follow-up durations, methods for diagnosing AF recurrence, number of patients with AF recurrence post-ablation, and covariates adjusted for in estimating the association between preprocedural SII and AF recurrence.

### Statistical analysis

The primary objective of this meta-analysis was to evaluate the association between preprocedural SII and AF recurrence following catheter ablation. This association was compared between patients categorized as having high vs low SII at baseline, employing risk ratios (RR) and corresponding 95% confidence intervals (CIs). The secondary objective was to assess the relationship between SII as a continuous variable and AF recurrence post-ablation, summarized as RR and 95% CIs per 100-unit increment of SII. When adjusted estimates were reported per 1-unit increment, the corresponding log-risk estimates and standard errors were multiplied by 100, assuming a linear relationship between SII and the log-risk of AF recurrence across the observed range. This rescaling was conducted prior to pooling to ensure consistency among studies. When necessary, RRs and standard errors were derived from reported CIs or *P*-values and subsequently log-transformed to stabilize variance and approximate normality [26]. For studies reporting odds ratios (ORs) obtained from logistic regression models, ORs were converted to RRs using established methodologies that account for the baseline recurrence risk in the reference group, when available, through the equation: RR = OR / [(1 -- P_o_) + (P_o_ × OR)], where P_o_ represents the baseline risk (event proportion) in the reference (low-SII) group [28]. For studies that reported hazard ratios (HRs) from Cox proportional hazards models, HRs were treated as numerically equivalent to RRs, consistent with standard practice in prognostic meta-analyses of time-to-event outcomes with comparable follow-up durations. Between-study heterogeneity was assessed using the Cochrane *Q* statistic and the I^2^ statistic [29], with thresholds of < 25%, 25%–75%, and > 75% interpreted as low, moderate, and high heterogeneity, respectively. Pooled estimates were generated using a random-effects model to account for inherent variability across studies [26]. For meta-analyses displaying significant heterogeneity (I^2^ > 75%), in addition to I^2^, between-study variance (τ^2^) was estimated, and 95% prediction intervals (PIs) were calculated for the main categorical analysis to reflect the potential range of effects that may be expected in future studies, accounting for between-study heterogeneity [26]. The robustness of the overall effect was evaluated through leave-one-out sensitivity analyses [30]. For the primary outcome, prespecified subgroup analyses were conducted to explore whether specific study characteristics influenced the observed associations. Stratifications included sample size, mean patient age, proportion of men, cutoff values for SII, ablation methods, follow-up duration, and study quality scores as assessed by the NOS. For continuous moderators, subgroups were formed using median values as cutoff points. Additionally, univariate meta-regression was performed to assess the relationship between study-level variables (e.g., sample size, mean age, proportion of men, proportion of PaAF, mean LAD at baseline, cutoff for SII, follow-up duration, and NOS score) and effect estimates [26]. Potential publication bias was assessed using funnel plot symmetry, visual inspection, and Egger’s regression test [31]. A two-sided *P*-value < 0.05 was deemed statistically significant. All analyses were conducted using RevMan (version 5.3; Cochrane Collaboration, Oxford, UK) and Stata (version 17.0; StataCorp, College Station, TX, USA).

## Results

### Study inclusion

**Figure 1.**
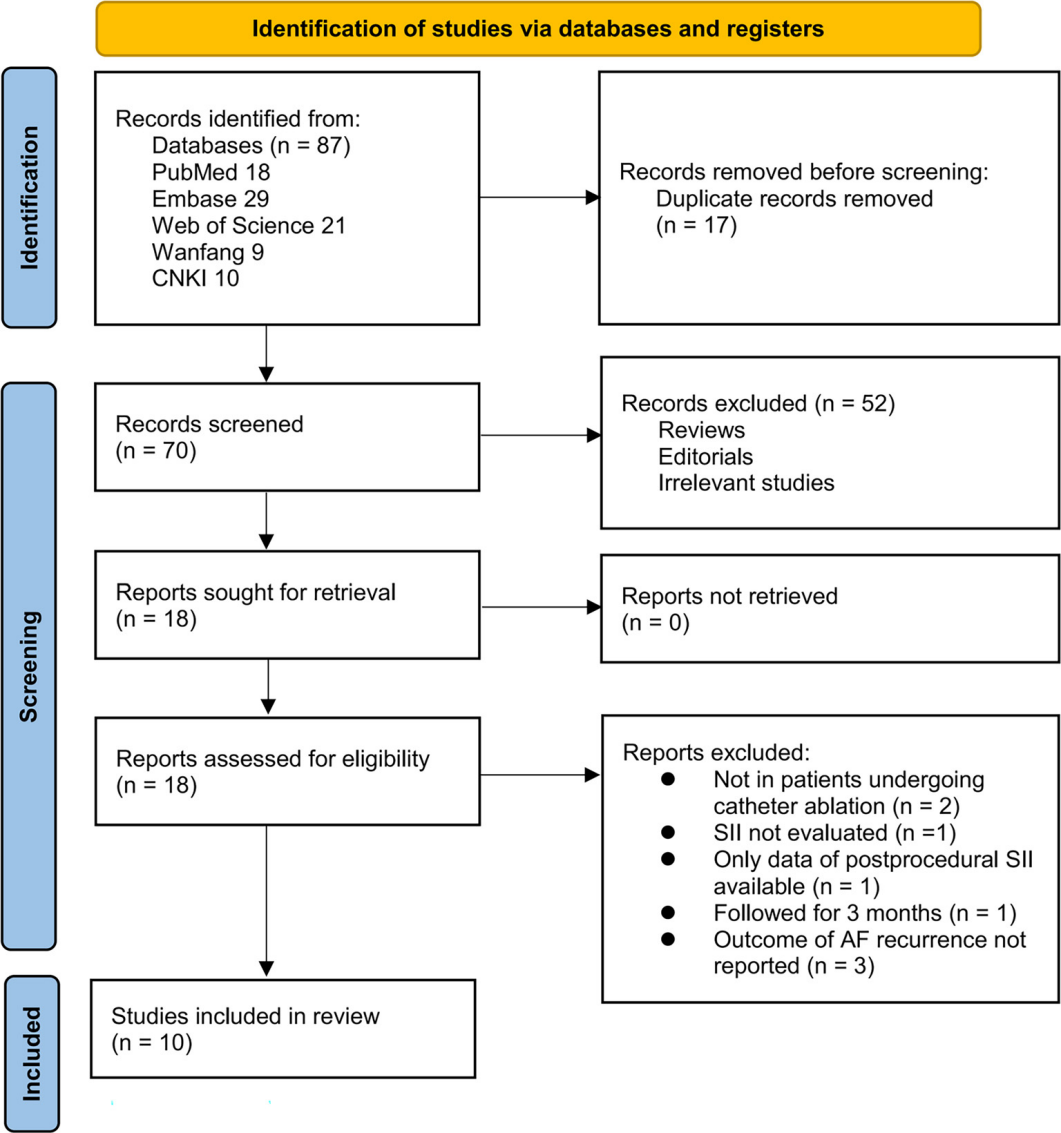
Flowchart of database search and study inclusion.

Figure 1 illustrates the study selection workflow. A total of 87 records were retrieved from five databases, with 17 duplicates eliminated. Screening of titles and abstracts led to the exclusion of 52 records that did not satisfy the eligibility criteria. The full texts of the remaining 18 articles were independently evaluated by two reviewers, resulting in the exclusion of eight articles for reasons detailed in Figure 1. Ultimately, 10 studies met all criteria and were included in the quantitative synthesis [15–24].

### Overview of the study characteristics

The key characteristics of the included studies are summarized in Table 1. A total of 10 retrospective cohort studies published between 2023 and 2025 were included, representing diverse geographical regions including China [16, 17, 19, 21–24], Turkey [15, 18], and Russia [20]. Sample sizes ranged from 204–757 patients, resulting in a cumulative total of 4,045 individuals with AF undergoing catheter ablation. Across studies, the mean age of participants varied from 55.0–65.3 years, and the proportion of men ranged from 39.2% to 78.9%. The proportion of PaAF was reported to range from 45.6% to 100% in seven studies [16–19, 22–24], while the mean baseline LAD varied between 37.5 and 42.8 mm in eight studies [15–19, 21, 23, 24]. All studies measured the SII preprocedurally, either at admission, within 24 h before the procedure, or during preoperative evaluation. SII was analyzed categorically in eight studies [15, 16, 18–20, 22–24] (high vs. low, with cutoff values ranging from 327.7–624.0, determined by ROC curve analysis) and as a continuous variable in seven studies [17, 18, 20–24]. Ablation methods included CBA in five studies [15, 17, 18, 20, 22], RFCA in four studies [16, 19, 21, 23], and a mixed RFCA/CBA approach in one study [24]. The mean follow-up duration ranged from 11.1–27.0 months, with most studies employing rhythm surveillance through scheduled electrocardiograms (ECGs) and 24-hour Holter monitoring at 1, 3, 6, and 12 months, supplemented by symptom-triggered evaluations. Importantly, all studies consistently defined AF recurrence as AF/AT/AFL lasting greater than 30 s occurring after the 3-month blanking period. The number of patients experiencing AF recurrence varied significantly across studies (from 63–448 cases), with a total of 1,279 (31.6%) patients experiencing AF recurrence during follow-up. All studies performed multivariable adjustments for key clinical factors, including age, sex, AF type, hypertension, LAD, renal function indices, left ventricular ejection fraction, inflammatory biomarkers, and stroke risk scores, although the extent of covariate adjustments varied among studies.

### Study quality evaluation

Study quality was assessed using the NOS, with total scores ranging from 7–9, indicating that all included studies were of high methodological quality (Table 2). Four studies [17, 18, 21, 23] achieved the maximum score of 9, reflecting strong cohort representativeness, clear exposure ascertainment, comprehensive confounder adjustment, standardized outcome assessment, and adequate follow-up. Three studies [15, 19, 22] scored 8, generally performing well across most domains but exhibiting minor limitations such as incomplete follow-up or cohort representativeness. The remaining studies [16, 20, 24] scored 7, primarily due to limited representativeness of the exposed cohort, less comprehensive follow-up procedures, or short follow-up duration. Across all studies, exposure ascertainment (preprocedural SII measurement) and outcome assessment (standardized rhythm monitoring) were consistently robust, and all studies controlled for age and at least one additional significant confounder. Collectively, the NOS results affirm that the included evidence base is generally reliable, with no studies rated as poor quality.

**Table 1:** Characteristics of the included studies

**Study**	**Country**	**Design**	**No. of patients with AF**	**Mean age (years)**	**Men (%)**	**PaAF (%)**	**LAD (mm)**	**Timing of SII measurement**	**SII analytic methods**	**Ablation method**	**Follow-up duration (months)**	**Validation for AF recurrence**	**Number of patients with AF recurrence**	**Variables adjusted**
Kaplan 2023	Turkey	RC	370	56.1	50.3	NR	39.0	24 hours before CBA	Categorical (High vs. Low). Cutoff determined by ROC curve analysis. Cutoff value: 532	CBA	25	ECG, 24-hour Holter ECG at scheduled visits (1, 3, 6, 12 months), and during symptom-related admissions. Recurrence defined as AF/AT/AFL >30 s after the 3-month blanking period	77	Age, CHA_2_DS_2_-VASc score, EHRA score, LVEF, and early recurrence
Zhao 2023	China	RC	204	55.0	78.9	45.6	41.0	Preoperative (at admission)	Categorical (High vs. Low). Cutoff determined by ROC curve analysis. Cutoff value: 444.8	RFCA	20	ECG and 24-hour Holter at 1, 3, and 6 months, then outpatient clinic or telephone follow-up. Recurrence defined as AF/AT/AFL >30 s after the 3-month blanking period	77	Age, Sex, BMI, hypertension, smoking, duration of AF, type of AF, CHA_2_DS_2_-VASc score, LAD, LVEF, HbA1c, eGFR, and hs-CRP
Kalenderoglu 2024	Turkey	RC	399	59.5	60.4	54.6	40.0	Preoperative (at admission)	Categorical (High vs. Low). Cutoff determined by ROC curve analysis. Cutoff value: 624. Continuous (per 1-unit increment)	CBA	27	Clinical symptoms, 12-lead ECG, and 24-hour Holter monitoring at 1, 3, 6, 12 months and annually thereafter. Recurrence defined as AF/AT/AFL >30 s after the 3-month blanking period	150	Age, hypertension, paroxysmal AF type, glucose, and LAD
Gu 2024	China	RC	307	65.3	53.1	56.0	42.8	Preoperative	Continuous (per 1-unit increment)	CBA	12	24-hour Holter monitoring at 1, 3, 6, and 12 months post-ablation. Recurrence defined as AF/AFL/AT >30 s after the 3-month blanking period	65	Age, persistent AF, NYHA class, SCr, LAD, and the other inflammatory indices
Wang 2024	China	RC	457	61.3	62.8	68.5	39.3	Preoperative (on the second day after admission, before RFCA)	Categorical (High vs. Low). Cutoff determined by ROC curve analysis. Cutoff value: 619.3	RFCA	12	Clinical assessment, 12-lead ECG, and 24-hour Holter monitoring at 1, 3, 6, and 12 months. Recurrence defined as AF/AFL/AT >30 s after the 3-month blanking period	113	Age, AF type, LAD, BNP, eGFR, and APPLE score
Basieva 2025	Russia	RC	239	61.0	53.0	NR	NR	Preoperative	Categorical (High vs. Low). Cutoff determined by ROC curve analysis. Cutoff value: 365.8. Continuous (per 1-unit increment)	CBA	20	Mixed methods: Telephone call, interview, ECG records (ambulance/hospital), Holter monitoring, hospitalization data. Recurrence defined as AF/AT/AFL >30 s after the 3-month blanking period	63	Age, sex, AF Duration, CAD, LAD, and use of class III antiarrhythmic drugs
Jie 2025	China	RC	757	60.2	60.0	68.0	NR	Preoperative (within 24 h preceding the procedure)	Categorical (High vs. Low). Cutoff determined by ROC curve analysis. Cutoff value: 327.7. Continuous (per 1-unit increment)	CBA	24.5	Outpatient clinic visits with 12-lead ECG and 24-hour Holter monitoring at 1, 3, 6, 12 months and every 6 months thereafter. Patients also used handheld ECG monitors for symptomatic episodes. Recurrence defined as AF/AT/AFL >30 s after the 3-month blanking period	448	Age, sex, BMI, persistent AF, smoking, alcohol, hypertension, CAD, NYHA class, CHA_2_DS_2_-VASc score, TC, TG, HDL-C, LDL-C, BNP, ARNI, rivaroxaban, statins, RBC count, WBC count, Hb, potassium, sodium, calcium, and LAD
Zhang 2025	China	RC	418	62.5	39.2	100.0	37.5	Preoperative	Categorical (High vs. Low). Cutoff determined by ROC curve analysis. Cutoff value: 579.5. Continuous (per 100-unit increment)	RFCA or CBA	11.1	Outpatient visits, phone calls, or social networking software at 3, 6, 12 months and every 6 months thereafter. Recurrence defined as AF/AT/AFL >30 s after the 3-month blanking period	75	Age, sex, BMI, smoking, DM, CAD, type of ablation (RFA/CBA), LAD, LVEF, NT-proBNP, eGFR, HbA1c, TG, and CRP
Nie 2025	China	RC	394	64.3	56.9	58.9	39.1	Preprocedural	Categorical (High vs. Low). Cutoff determined by ROC curve analysis. Cutoff value: 507.2. Continuous (per 1-unit increment)	RFCA	12	Clinical evaluation, 12-lead ECG, and 24-hour Holter monitoring at 1, 3, 6, and 12 months. Symptomatic patients had additional ECGs/Holter. Recurrence defined as AF/AT/AFL >30 s after the 3-month blanking period	88	Age, SCr, non-paroxysmal AF, HF, LAD, LVEF, and BNP
Guo 2025	China	RC	500	63.0	59.2	NR	41.5	Preoperative (at admission)	Continuous (per 1-unit increment)	RFCA	12	ECG and 24-hour Holter monitoring were used to document recurrence during follow-up. Recurrence defined as AF/AT/AFL >30 s after the 3-month blanking period	123	Age, sex, LAD, LVEF, CRP, NT-proBNP, UA, SCr, TG, and CK-MB

Abbreviations: AF: Atrial fibrillation; AFL: Atrial flutter; APPLE: Age–persistent AF–impaired renal function–left atrial diameter–EF score; ARNI: Angiotensin receptor–neprilysin inhibitor; AT: Atrial tachycardia; BMI: Body mass index; BNP: B-type natriuretic peptide; CBA: Cryoballoon ablation; CAD: Coronary artery disease; CK-MB: Creatine kinase–myocardial band; DM: Diabetes mellitus; ECG: Electrocardiogram; eGFR: Estimated glomerular filtration rate; EHRA: European Heart Rhythm Association; Hb: Hemoglobin; HbA1c: Glycated hemoglobin; HDL-C: High-density lipoprotein cholesterol; hs-CRP: High-sensitivity C-reactive protein; LAD: Left atrial diameter; LDL-C: Low-density lipoprotein cholesterol; LVEF: Left ventricular ejection fraction; NR: Not reported; NT-proBNP: N-terminal pro-B-type natriuretic peptide; NYHA: New York Heart Association; PaAF: Paroxysmal atrial fibrillation; RC: Retrospective cohort; RBC: Red blood cell; RFCA: Radiofrequency catheter ablation; ROC: Receiver operating characteristic; SCr: Serum creatinine; SII: Systemic immune-inflammation index; TC: Total cholesterol; TG: Triglyceride; UA: Uric acid; WBC: White blood cell.

**Table 2:** Evaluation of study quality using the Newcastle-Ottawa Scale

**Study**	**Representativeness of the exposed cohort**	**Selection of the non-exposed cohort**	**Ascertainment of exposure**	**Outcome not present at baseline**	**Control for age**	**Control for other confounding factors**	**Assessment of outcome**	**Enough long follow-up duration**	**Adequacy of follow-up of cohorts**	**Total**
Kaplan 2023	1	1	1	1	1	1	1	1	0	8
Zhao 2023	0	1	1	1	1	1	1	1	0	7
Kalenderoglu 2024	1	1	1	1	1	1	1	1	1	9
Gu 2024	1	1	1	1	1	1	1	1	1	9
Wang 2024	0	1	1	1	1	1	1	1	1	8
Basieva 2025	0	1	1	1	1	1	1	1	0	7
Jie 2025	0	1	1	1	1	1	1	1	1	8
Zhang 2025	0	1	1	1	1	1	1	0	1	7
Nie 2025	1	1	1	1	1	1	1	1	1	9
Guo 2025	1	1	1	1	1	1	1	1	1	9

### Meta-analysis results with SII analyzed as categorized variables

**Figure 2.**
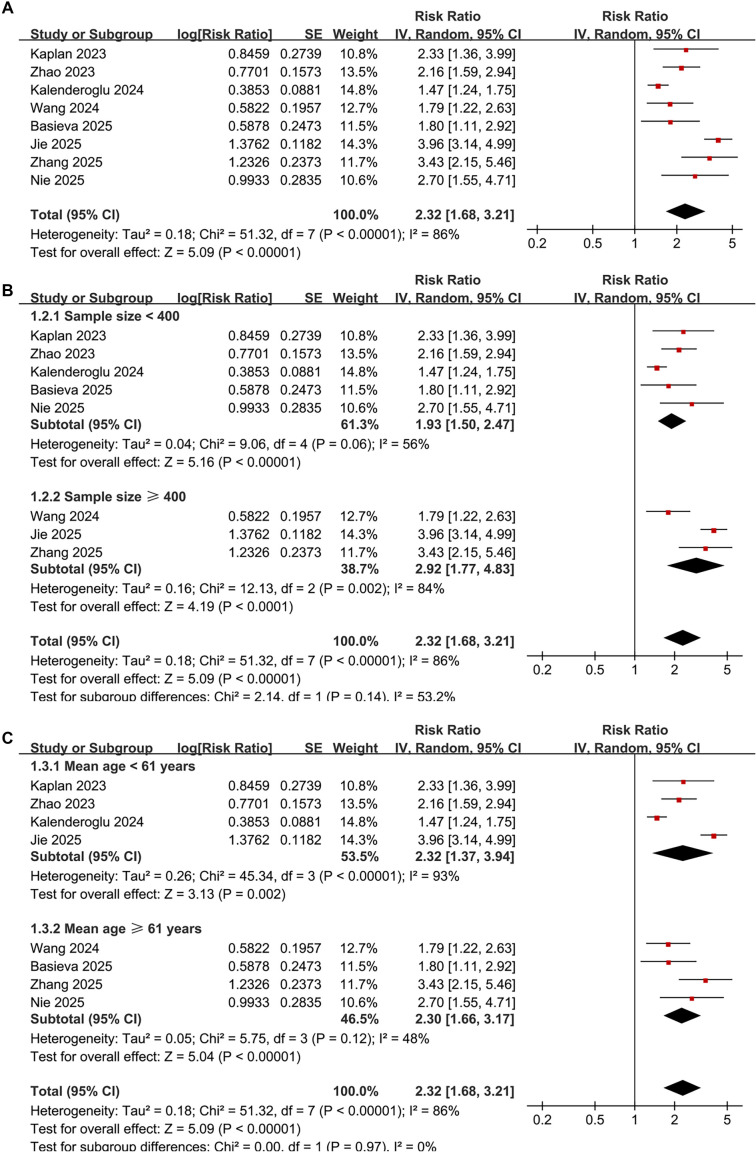
**Forest plots illustrating the risk of atrial fibrillation recurrence after catheter ablation based on high versus low preprocedural systemic immune-inflammation index.** (A) Overall inverse-variance random-effects meta-analysis of eight cohort studies shows that high preprocedural SII is associated with a higher risk of post-ablation AF recurrence (pooled RR = 2.32, 95% CI 1.68–3.21; *P* < 0.001), with substantial heterogeneity (τ^2^ ═ 0.18; I^2^ ═ 86%; Cochran *Q* *P* < 0.01). (B) Subgroup analysis by study sample size demonstrates similar associations in studies with *n* < 400 (RR = 1.93, 95% CI 1.50–2.47) and *n*≥ 400 (RR = 2.92, 95% CI 1.77–4.83), with no evidence of a difference between subgroups (*P* for subgroup difference = 0.14). (C) Subgroup analysis by mean participant age shows consistent associations in studies with mean age < 61 years (RR = 2.32, 95% CI 1.37–3.94) and ≥ 61 years (RR = 2.30, 95% CI 1.66–3.17), with no evidence of a difference between subgroups (*P* for subgroup difference = 0.97). In all panels, squares represent study-specific RRs (area proportional to study weight), horizontal lines indicate 95% CIs, and diamonds denote pooled effects; the vertical line indicates no association (RR = 1) and the x-axis is plotted on a logarithmic scale. Abbreviations: AF: Atrial fibrillation; CI: Confidence interval; I^2^: I-squared heterogeneity statistic; RR: Risk ratio; SE: Standard error; SII: Systemic immune–inflammation index; τ^2^: Between-study variance; *Q*: Cochran’s *Q*.

Pooled results from eight studies [15, 16, 18–20, 22–24] analyzing the SII as a categorical variable indicated that patients with high preprocedural SII have a significantly increased risk of AF recurrence following catheter ablation compared to those with low SII (RR: 2.32, 95% CI: 1.68–3.21, *P*< 0.001; Figure 2A). Notably, there was significant heterogeneity among the studies (*P* for Cochrane *Q* test < 0.01; I^2^ ═ 86%; τ^2^ ═ 0.18). The corresponding 95% prediction interval ranged from 1.01–5.33, suggesting considerable variability in the observed associations across different clinical contexts. Sensitivity analyses, excluding one study at a time, yielded consistent results (RR: 2.07–2.53, *P* all < 0.05). Furthermore, the exclusion of a study that adjusted for early recurrence—a post-ablation variable—[15] resulted in a stable pooled estimate, indicating that the association was not significantly affected by potential overadjustment (RR: 2.32, 95% CI: 1.62–3.31, *P*< 0.001; I^2^ ═ 88%).

**Figure 3.**
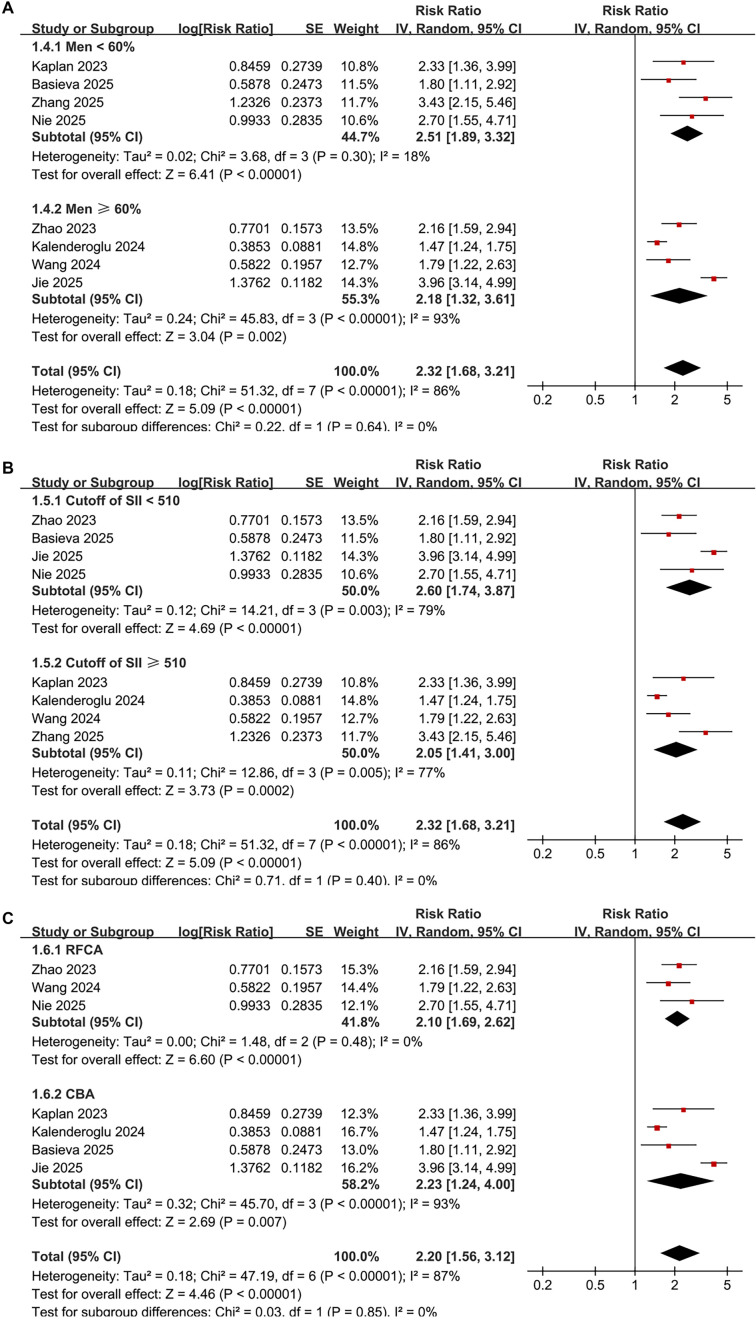
**Forest plots for subgroup analyses comparing atrial fibrillation recurrence after catheter ablation in patients with high versus low preprocedural systemic immune–inflammation index.** (A) Subgroup analysis stratified by the proportion of men (<60% vs ≥60%) shows consistent associations (RR = 2.51 vs 2.18; *P* for subgroup difference = 0.64). (B) Subgroup analysis stratified by the study-specific cutoff used to define high systemic immune–inflammation index (<510 vs ≥510) also shows similar effects (RR = 2.60 vs 2.05; *P* for subgroup difference = 0.40). (C) Subgroup analysis stratified by ablation modality demonstrates comparable associations in radiofrequency catheter ablation and cryoballoon ablation cohorts (RR = 2.10 vs 2.23; *P* for subgroup difference = 0.85); the mixed-modality study (Zhang 2025), which included both modalities without modality-specific estimates, was excluded from this panel. In all panels, squares represent study-specific risk ratios (size proportional to inverse-variance weight), horizontal lines indicate 95% confidence intervals, diamonds denote pooled effects from inverse-variance random-effects models, and the vertical line indicates no association (RR = 1) on a logarithmic scale. Abbreviations: AF: Atrial fibrillation; CBA: Cryoballoon ablation; CI: Confidence interval; RFCA: Radiofrequency catheter ablation; RR: Risk ratio; SII: Systemic immune–inflammation index.

**Figure 4.**
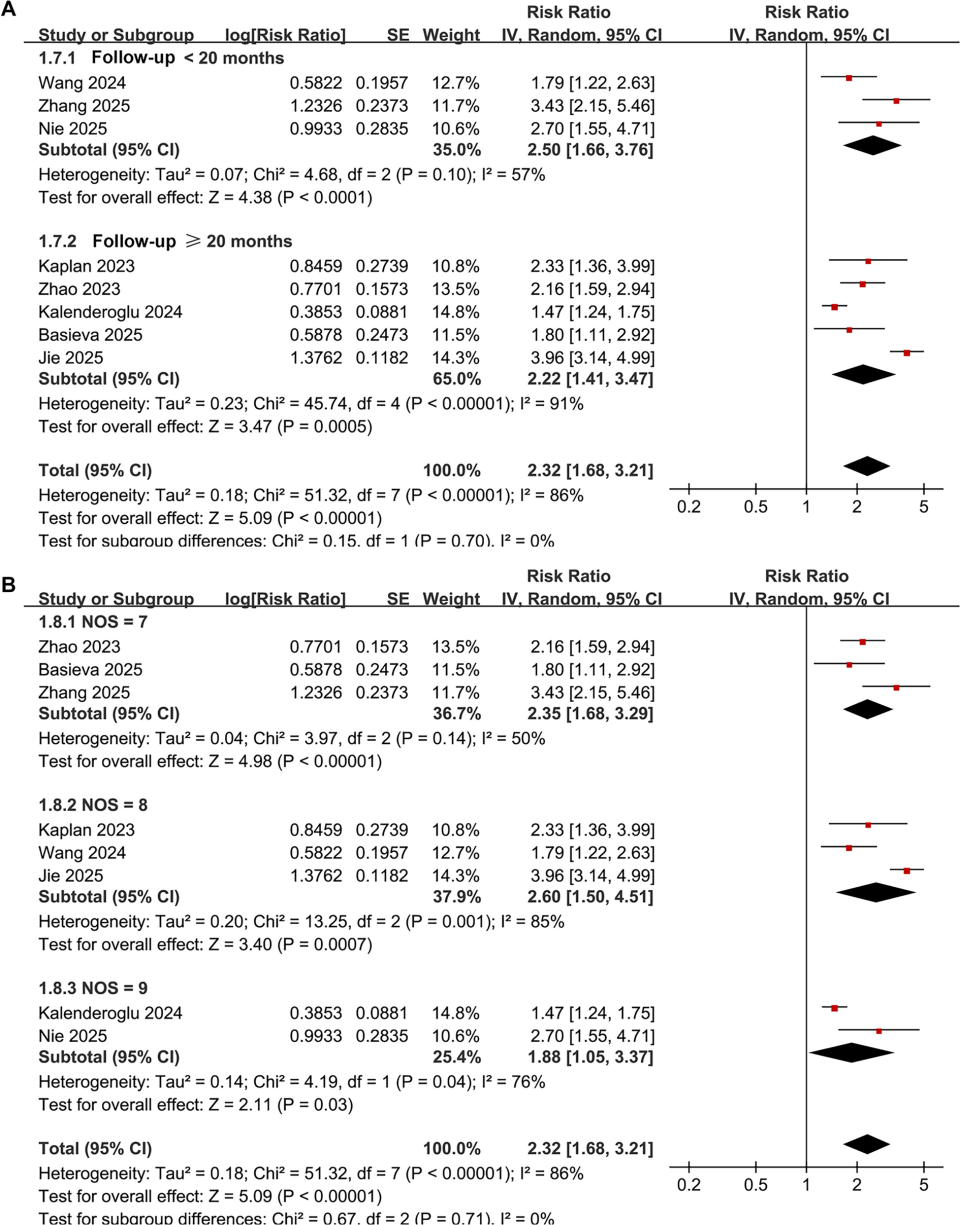
**Forest plots for subgroup analyses comparing atrial fibrillation recurrence after catheter ablation in patients with high versus low preprocedural systemic immune–inflammation index.** (A) Subgroup analysis stratified by mean follow-up duration (<20 vs ≥20 months) shows a consistent association between high preprocedural SII and AF recurrence, with pooled estimates of RR = 2.50 (95% CI 1.66–3.76) for follow-up <20 months and RR = 2.22 (95% CI 1.41–3.47) for follow-up ≥20 months; no evidence of a subgroup difference was observed (*P* for subgroup difference = 0.70). (B) Subgroup analysis stratified by study quality (Newcastle–Ottawa Scale score 7, 8, or 9) demonstrates broadly comparable associations across quality strata, with no evidence of a subgroup difference (*P* for subgroup difference = 0.71). In both panels, squares represent study-specific risk ratios (size proportional to inverse-variance weight), horizontal lines indicate 95% confidence intervals, and diamonds denote pooled effects from inverse-variance random-effects models; the vertical line indicates no association (RR = 1) on a logarithmic scale. Abbreviations: AF: Atrial fibrillation; CI: Confidence interval; NOS: Newcastle–Ottawa Scale; RR: Risk ratio; SII: Systemic immune–inflammation index.

Subgroup analyses produced similar results across varying sample sizes (< 400 or ≥ 400) (RR: 1.93 vs. 2.92, *P* for subgroup difference = 0.14; Figure 2B), and in patients stratified by mean ages (< 61 or ≥ 61 years) (RR: 2.32 vs. 2.30, *P* for subgroup difference = 0.97; Figure 2C). The results remained consistent across studies with male proportions (< 60% or ≥ 60%) (RR: 2.51 vs. 2.18, *P* for subgroup difference = 0.64; Figure 3A), varying cutoffs for defining high SII (< 510 or ≥ 510) (RR: 2.60 vs. 2.05, *P* for subgroup difference = 0.40; Figure 3B), and among patients receiving RFCA and CBA (RR: 2.10 vs. 2.23, *P* for subgroup difference = 0.85; Figure 3C). Additionally, a consistent association between SII and AF recurrence was observed across studies with follow-up durations of < 20 months and ≥ 20 months (RR: 2.50 vs. 2.22, *P* for subgroup difference = 0.70; Figure 4A), as well as among studies of varying quality scores (*P* for subgroup difference = 0.71; Figure 4B).

Univariate meta-regression analysis did not identify any study-level characteristics that significantly modified the association between preprocedural SII and the risk of AF recurrence post-ablation (Table 3). Variations in sample size, mean age, proportion of men, prevalence of PaAF, and mean LAD did not yield meaningful changes in effect size (all *P* > 0.10). Similarly, differences in SII cutoff values, follow-up duration, and study quality (measured by NOS score) were not statistically significant moderators of pooled risk estimates (all *P* > 0.10). Although some variables, such as sample size and SII cutoff, exhibited modest adjusted R^2^ values (30.3% and 31.0%, respectively), these trends did not achieve statistical significance (*P* ═ 0.13 and 0.16, respectively). Overall, meta-regression findings suggest that the observed relationship between elevated SII and increased AF recurrence risk is consistent across studies, with no clear influence from demographic factors, clinical characteristics, or methodological features.

**Table 3:** Results of univariate meta-regression analysis

**Variables**	**RR for the association between preprocedural SII and recurrence of AF after catheter ablation**			
	**Coefficient**	**95% CI**	***P* values**	**Adjusted R^2^**
Sample size	0.0012	--0.0005 to 0.0029	0.13	30.3%
Mean age (years)	0.026	--0.097 to 0.149	0.63	0%
Men (%)	--0.0096	--0.0417 to 0.0225	0.49	0%
PaAF (%)	0.012	--0.015 to 0.039	0.29	12.4%
Mean LAD (mm)	--0.17	--0.49 to 0.15	0.22	20.0%
Cutoff of SII	--0.0017	--0.0043 to 0.0009	0.16	31.0%
Follow-up duration (months)	--0.011	--0.069 to 0.047	0.66	0%
NOS	--0.10	--0.54 to 0.33	0.58	0%

Abbreviations: AF: Atrial fibrillation; CI: Confidence interval; LAD: Left atrial diameter; NOS: Newcastle–Ottawa Scale; PaAF: Paroxysmal atrial fibrillation; RR: Risk ratio; SII: Systemic immune-inflammation index.

**Figure 5.**
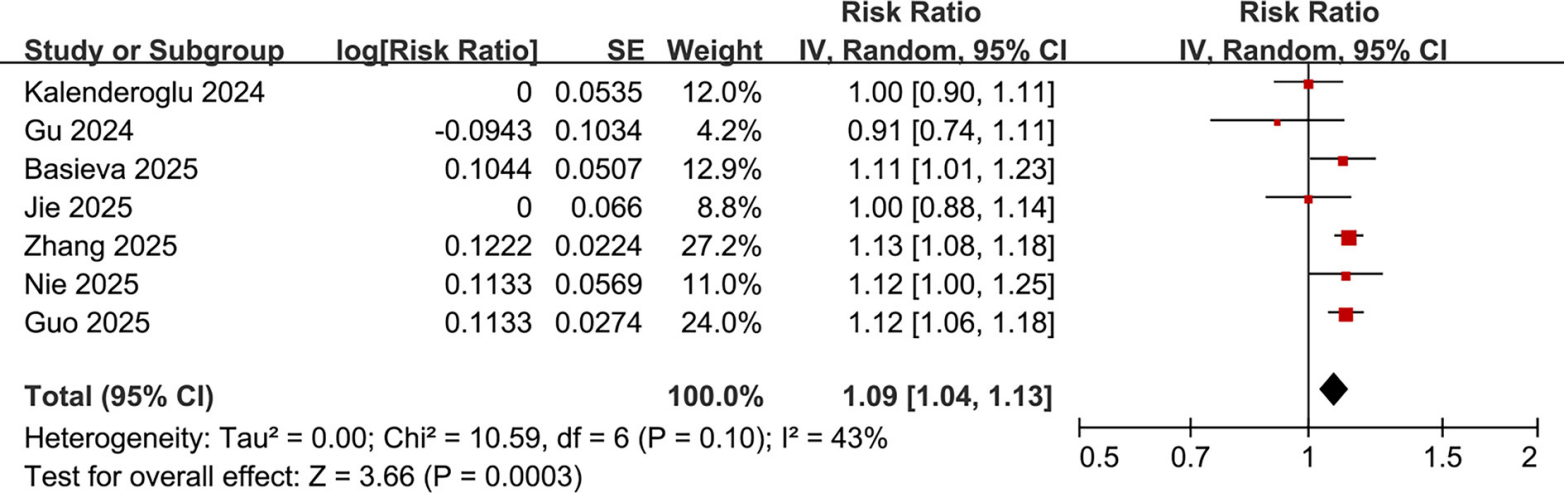
**Forest plot for the association between preprocedural systemic immune–inflammation index analyzed as a continuous variable and atrial fibrillation recurrence after catheter ablation.** Study-specific and pooled RRs are expressed per 100-unit increase in SII and were synthesized using an inverse-variance random-effects model. The pooled analysis of seven cohort studies shows that higher baseline SII is associated with an increased risk of post-ablation AF recurrence (pooled RR per 100-unit increment = 1.09, 95% CI 1.04–1.13; *P* < 0.001), with moderate heterogeneity (I^2^ ═ 43%; Cochran *Q* *P* ═ 0.10). Squares represent study-specific estimates (size proportional to inverse-variance weight), horizontal lines indicate 95% confidence intervals, and the diamond denotes the pooled effect; the vertical line indicates no association (RR = 1) and the x-axis is plotted on a logarithmic scale. Abbreviations: AF: Atrial fibrillation; CI: Confidence interval; RR: Risk ratio; SII: Systemic immune–inflammation index.

### Meta-analysis results with SII analyzed as continuous variables

Pooled results from seven studies [17, 18, 20–24] that analyzed SII as a continuous variable similarly indicated that higher baseline SII is associated with an increased risk of AF recurrence following catheter ablation (RR per 100-unit increment of SII: 1.09, 95% CI: 1.04–1.13, *P*< 0.001; Figure 5) with moderate heterogeneity (*P* for Cochrane *Q* test = 0.10; I^2^ ═ 43%). Sensitivity analyses, excluding one study at a time, did not significantly alter the results (RR: 1.07–1.10, *P* all < 0.05).

### Publication bias

**Figure 6.**
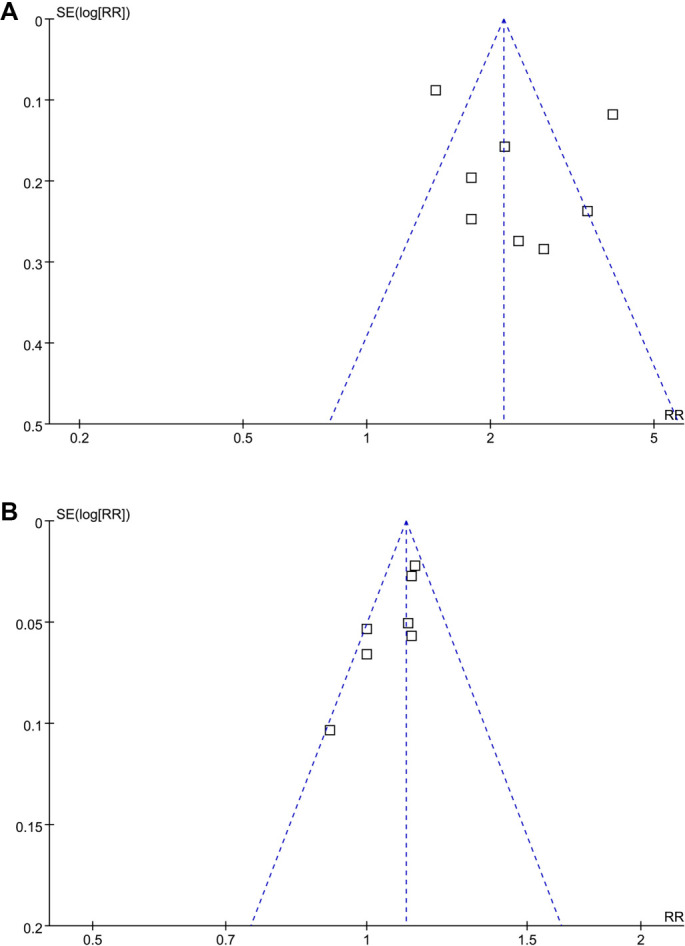
**Funnel plots assessing potential publication bias for the meta-analyses of preprocedural systemic immune–inflammation index and atrial fibrillation recurrence after catheter ablation.** (A) Categorical analysis (high vs low SII). (B) Continuous analysis (risk per 100-unit increase in SII). Each square represents an individual study; the y-axis shows the standard error of log(RR). The dashed vertical line indicates the pooled effect and the dashed diagonal lines show the 95% pseudo–confidence limits. Abbreviations: AF: Atrial fibrillation; RR: Risk ratio; SE: Standard error; SII: Systemic immune–inflammation index.

Figures 6A and 6B display funnel plots assessing potential publication bias for the meta-analyses of SII analyzed as categorical and continuous variables. Given that only seven to eight studies contributed to each analysis, the statistical power to detect small-study effects is limited, and both visual inspection and formal tests should be interpreted cautiously. No significant asymmetry was evident on visual inspection, and Egger’s regression tests were not statistically significant (*P* ═ 0.42 for the categorical analysis and *P* ═ 0.21 for the continuous analysis), although these findings do not exclude the possibility of publication bias.

## Discussion

This meta-analysis demonstrates that a higher preprocedural SII is consistently associated with an elevated risk of AF recurrence following catheter ablation. By synthesizing evidence from ten cohort studies encompassing over 4,000 patients and integrating both categorical and continuous analyses while exploring heterogeneity across clinically relevant subgroups, this study offers a comprehensive and current synthesis of available evidence regarding preprocedural SII and AF recurrence post-ablation. The findings were robust across sensitivity analyses, stable when individual studies were sequentially excluded, and reproducible across a wide range of prespecified subgroups. Moreover, analyses treating SII as a continuous variable revealed a graded linear association, with higher SII values correlating with progressively increased risk of AF recurrence per 100-unit increment. Collectively, these results support SII as a clinically significant indicator of preprocedural inflammatory status associated with ablation outcomes.

Several biological mechanisms may underlie the observed association. SII integrates three circulating components—neutrophils, platelets, and lymphocytes—each implicated in AF pathogenesis [32, 33]. Elevated neutrophil counts contribute to oxidative stress, the release of proteolytic enzymes, and inflammatory cytokine signaling, all of which promote atrial myocyte injury, conduction heterogeneity, and structural remodeling [34]. Platelets are involved not only in thrombogenesis but also in immune activation, secreting pro-inflammatory mediators that enhance leukocyte recruitment and endothelial dysfunction [35]. Decreased lymphocyte counts, indicative of relative immunosuppression or heightened inflammatory burden, may impair regulatory immune pathways that counteract inflammation-driven arrhythmogenic remodeling [36, 37]. Collectively, a high SII reflects a state of increased neutrophil- and platelet-mediated inflammation combined with diminished adaptive immune regulation, potentially facilitating the persistence of substrate abnormalities even after technically successful ablation. This biological environment favors reconnection of pulmonary veins, non-pulmonary vein triggers, and ongoing atrial remodeling, thereby heightening the likelihood of recurrent AF [38].

The consistency of the association across various subgroups further enhances the reliability of these findings. The relationship between high SII and recurrence remained similar regardless of sample size, mean age, sex distribution, or prevalence of PaAF, suggesting that the prognostic value of SII is not confined to a specific demographic profile. Likewise, comparable associations across different SII cutoff values and follow-up durations indicate that the effect is robust against methodological variations. Notably, the association was observed in both RFCA and CBA cohorts, implying that inflammatory vulnerability may influence recurrence risk independently of energy modality. Sensitivity analyses demonstrated minimal fluctuation in overall effect estimates, further reinforcing the stability of the conclusion. Although meta-regression did not identify statistically significant effect modifiers, trends were noted for sample size and SII cutoff, each accounting for approximately 30% of the between-study heterogeneity. While these trends did not achieve significance, they suggest that variability in inflammation thresholds or study precision may modestly contribute to differences in effect size, warranting further investigation utilizing individual participant data (IPD). Conversely, meta-regression analyses did not reveal significant study-level modifiers of the association between preprocedural SII and AF recurrence, indicating that the substantial heterogeneity observed could not be attributed to the available aggregated variables. This unexplained heterogeneity may reflect unmeasured or inconsistently reported factors, such as differences in comorbidity burden, medication use, inflammatory comorbidities, procedural techniques, operator experience, rhythm-monitoring intensity, timing of SII measurement, and residual confounding within individual studies. Additionally, the limited number of included studies restricts the statistical power of meta-regression to detect genuine effect modification. Consequently, the absence of significant moderators should not be interpreted as evidence that these factors are unimportant, but rather as an inherent limitation of study-level analyses.

This study has several strengths. The search was comprehensive and current, encompassing both international and major Chinese databases. All included studies employed cohort designs with longitudinal follow-up, and nearly all implemented multivariable adjustments to mitigate confounding, thereby enhancing the credibility of the pooled estimates. The analyses utilized both categorical and continuous SII metrics and employed multiple sensitivity and subgroup strategies to ensure robustness. The inclusion of meta-regression provided additional insights into sources of heterogeneity, and consistent findings across analytic approaches reinforced the reliability of the observed associations.

However, several limitations must be considered when interpreting these results. First, all included studies were retrospective cohorts, which may introduce recall bias, selection bias, and residual confounding despite adjustments [39]. Additionally, these studies were conducted in China, Turkey, and Russia, potentially limiting the generalizability of the findings to other regions, ethnic groups, and healthcare systems, especially where patient characteristics, inflammatory profiles, and ablation practices differ. Second, substantial heterogeneity was present, likely reflecting differences in patient demographics, comorbidities, baseline AF burden, ablation strategies, operator experience, SII measurement timing, and SII cutoff values. These factors could not be explored in depth due to the lack of IPD, limiting the precision of subgroup analyses and the ability to fully adjust for clinically relevant modifiers. The substantial heterogeneity (I^2^ ═ 86%) and the wide 95% prediction interval indicate that the pooled RR represents an average association across heterogeneous studies, and the true effect in an individual clinical setting may vary considerably, ranging from minimal to substantially increased recurrence risk, thus limiting direct clinical extrapolation. Notably, all studies defined “high SII” using ROC curve-derived cutoffs, which may introduce optimism bias and inflate effect estimates, particularly in retrospective contexts. Since no study applied externally validated or prespecified thresholds, stratification by cutoff derivation was not feasible, and subgroup analyses based on cutoff magnitude were restricted by small sample sizes and low statistical power. Consequently, these analyses should be regarded as exploratory rather than confirmatory. One included study adjusted for early recurrence [15], which may represent an intermediate variable on the causal pathway to later recurrence. However, sensitivity analysis excluding this study yielded consistent results, suggesting minimal impact from potential overadjustment.

Third, although multivariable analyses were conducted, unmeasured or inconsistently reported confounders cannot be excluded, including medications, inflammation-modifying comorbidities, and peri-procedural management differences. Moreover, subgroup analyses and meta-regressions were based on a limited number of studies and should therefore be regarded as exploratory; interaction *P*-values were reported, but these analyses were underpowered to detect true effect modification, and the absence of statistically significant interactions should not be interpreted as evidence of homogeneity. Additionally, although AF is a key determinant of ablation success, stratified analyses by paroxysmal vs persistent AF could not be performed because most included studies did not report effect estimates separately by AF type, thus limiting the assessment of effect modification. Fourth, causality cannot be inferred as this meta-analysis is based on observational studies. Elevated SII may reflect an underlying predisposition to arrhythmogenic remodeling rather than serving as a direct contributor to recurrence. Furthermore, while AF recurrence was consistently defined across studies, rhythm surveillance strategies varied significantly, including differences in scheduled ECG/Holter monitoring, symptom-triggered assessments, and telephone or mixed follow-up methods, which may have influenced recurrence detection and contributed to residual heterogeneity. Although standard approaches were used to harmonize ORs and HRs to the RR scale, some degree of approximation is unavoidable and may contribute modestly to residual heterogeneity. Similarly, the continuous analysis assumes linearity between SII and log-risk, which is consistent with the original modeling approaches but cannot fully exclude potential nonlinear effects. Additionally, the absence of grey literature and trial registries in the search may increase susceptibility to publication bias, though this approach prioritized peer-reviewed evidence with more reliable methodological reporting. While publication bias cannot be entirely ruled out given the modest number of available studies, funnel plots and Egger’s tests did not indicate significant asymmetry. Finally, absolute recurrence risks or absolute risk differences could not be reliably presented because SII categories were defined using heterogeneous, study-specific cutoffs, and most studies reported adjusted relative estimates without providing stratified baseline risks.

From a clinical perspective, these findings suggest that SII may serve as a simple and widely accessible biomarker to assist in preprocedural risk stratification for patients undergoing catheter ablation. Prior research has demonstrated that elevated SII reflects heightened systemic inflammation and is associated with various adverse AF-related outcomes. For instance, higher SII has been linked to left atrial thrombosis in nonvalvular AF, indicating its relevance to atrial substrate vulnerability and prothrombotic inflammatory activation [40]. SII has also shown prognostic value in identifying patients at increased risk of recurrence following cryo-Maze surgery and direct current cardioversion [41], underscoring its potential utility across different rhythm-control strategies beyond catheter ablation [42]. Additionally, elevated SII has been associated with systemic inflammatory states contributing to left atrial appendage dysfunction, which may further predispose individuals to arrhythmia persistence or recurrence. Although one recent study reported no association between SII and post-ablation pericarditis, the authors emphasized that pericarditis reflects a distinct acute inflammatory response rather than a chronic inflammatory milieu relevant to AF recurrence, suggesting that SII may still hold predictive value in the long-term arrhythmogenic context rather than peri-procedural complications [43]. Taken together, these studies provide mechanistic and clinical support for the role of inflammation—captured by SII—in AF-related outcomes and complement the present meta-analytic finding that elevated SII precedes arrhythmia recurrence. Nevertheless, given the inherent limitations of observational evidence and the heterogeneity across available studies, SII should not be used in isolation to guide clinical decision-making. Instead, it may be incorporated as part of a broader clinical profile to help identify individuals with heightened inflammatory susceptibility who could benefit from closer rhythm surveillance or more intensive risk-factor optimization. Future prospective studies are needed to standardize SII measurement, validate optimal thresholds, clarify its incremental prognostic value relative to established predictors, and examine temporal changes in inflammatory markers before and after ablation. IPD meta-analyses may further elucidate how inflammation interacts with demographic factors, procedural characteristics, and comorbidities in shaping recurrence risk.

## Conclusion

In conclusion, this meta-analysis indicates that higher preprocedural SII is associated with an increased likelihood of AF recurrence after catheter ablation. This association is accompanied by substantial heterogeneity and is largely based on study-specific, ROC-derived thresholds that may overestimate prognostic effects. Accordingly, SII should be viewed as a potential adjunct marker reflecting inflammatory vulnerability, complementing established clinical and procedural risk factors rather than serving as a standalone predictor. Prospective studies using standardized or externally validated SII thresholds are required to determine its incremental prognostic value and clarify its role in clinical risk stratification.

## Supplemental data

**Supplemental file 1.** Detailed search strategy for each database

**PubMed**

(“systemic inflammation index”[tiab] OR “systemic immune-inflammation index”[tiab] OR “systemic immune inflammatory index”[tiab] OR “systemic immune-inflammatory index”[tiab] OR “systemic-immune-inflammation index”[tiab] OR SII[tiab]) AND (“Atrial Fibrillation”[Mesh] OR “atrial fibrillation”[tiab] OR AF[tiab]) AND (“Catheter Ablation”[Mesh] OR “Ablation Techniques”[Mesh] OR “Electrophysiologic Techniques, Cardiac”[Mesh] OR “Catheters”[Mesh] OR ablation*[tiab] OR catheter*[tiab] OR radiofrequency[tiab] OR RFCA[tiab] OR cryoballoon*[tiab] OR “pulmonary vein isolation”[tiab] OR PVI[tiab]) AND (“Recurrence”[Mesh] OR recurren*[tiab])

**Embase**

(’systemic inflammation index’:ti,ab,kw OR ’systemic immune-inflammation index’:ti,ab,kw OR ’systemic immune inflammatory index’:ti,ab,kw OR ’systemic immune-inflammatory index’:ti,ab,kw OR ’systemic-immune-inflammation index’:ti,ab,kw OR sii:ti,ab,kw) AND (’atrial fibrillation’/exp OR ’atrial fibrillation’:ti,ab,kw OR af:ti,ab,kw) AND (’catheter ablation’/exp OR ’ablation’/exp OR ’pulmonary vein isolation’/exp OR ablation*:ti,ab,kw OR catheter*:ti,ab,kw OR radiofrequency:ti,ab,kw OR rfca:ti,ab,kw OR cryoballoon*:ti,ab,kw OR ’pulmonary vein isolation’:ti,ab,kw OR pvi:ti,ab,kw) AND (’recurrence’/exp OR recurren*:ti,ab,kw)

**Web of Science**

TS ═ ((“systemic inflammation index” OR “systemic immune-inflammation index” OR “systemic immune inflammatory index” OR “systemic immune-inflammatory index” OR “systemic-immune-inflammation index” OR SII) AND (“atrial fibrillation” OR AF) AND (ablation* OR catheter* OR radiofrequency OR RFCA OR cryoballoon* OR “pulmonary vein isolation” OR PVI) AND (recurren*))

**Wanfang**

(“
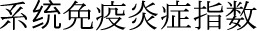
” OR “
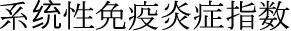
” OR SII OR ”systemic immune-inflammation index” OR ”systemic inflammation index”) AND (“
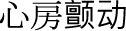
” OR “
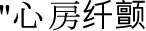
” OR “
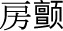
” OR ”atrial fibrillation” OR AF) AND (“
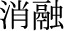
” OR “
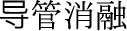
” OR “
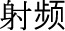
” OR “
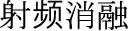
” OR “
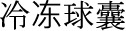
” OR “
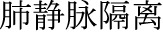
” OR PVI OR RFCA OR cryoballoon* OR ablation*) AND (“
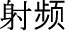
” OR “
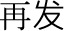
” OR recurrence)

**CNKI**

(“
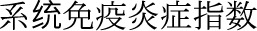
” OR “
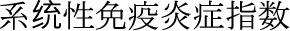
” OR SII OR ”systemic immune-inflammation index” OR ”systemic inflammation index”) AND (“
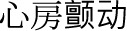
” OR “
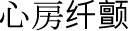
” OR “
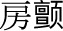
” OR ”atrial fibrillation” OR AF) AND (“
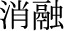
” OR “
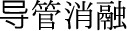
” OR “
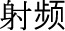
” OR “
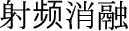
” OR “
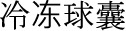
” OR “
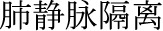
” OR PVI OR RFCA OR cryoballoon* OR ablation*) AND (“
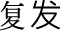
” OR “
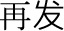
” OR recurrence)

## Data Availability

All data generated or analyzed during this study are included in this published article.
